# Three-dimensional-printed upper limb prosthesis for a child with traumatic amputation of right wrist: A case report

**DOI:** 10.1097/MD.0000000000009426

**Published:** 2017-12-29

**Authors:** Guisheng Xu, Liang Gao, Ke Tao, Shengxiang Wan, Yuning Lin, Ao Xiong, Bin Kang, Hui Zeng

**Affiliations:** a Department of Orthopaedics, Zhaoqing First People's Hospital, Guangdong, China; b Center of Experimental Orthopaedics, Saarland University, Homburg/Saar, Germany; c Department of Orthopaedics, Peking University People's Hospital, Peking; d Department of Orthopaedics, Peking University Shenzhen Hospital, Shenzhen, China.

**Keywords:** 3D printing, children, rehabilitation, upper limb prosthesis, wrist disarticulation

## Abstract

**Rationale:**

For traumatic upper limb amputees, the prohibitive cost of a custom-made prosthesis brings an insufferable financial burden for their families in developing countries. Three-dimensional (3D) printing allows for creating affordable, lightweight, customized, and well-fitting prosthesis, especially for the growing children.

**Patient concerns:**

We presented a case of an 8-year-old boy, who suffered a traumatic right wrist amputation as result of a mincing machine accident. The patient was immediately sent to the emergency orthopedics department after the accident.

**Diagnoses:**

He was diagnosed as severed mangled limb crash injury at the level of the right wrist with a Mangled Extremity Severity Score of 8.

**Interventions:**

A wrist disarticulation was performed and a 3D-printed prosthetic hand was designed and manufactured for this child. A personalized prosthetic rehabilitation training was applied after the prosthesis installation at 6 months postoperatively. The function of the prosthesis was evaluated at 1-month and 3-month follow-up using the Children Amputee Prosthetics Projects (CAPP) score and the University Of New Brunswick Test Of Prosthetic Function for Unilateral Amputees (UNB test).

**Outcomes:**

The materials cost <20 dollars. The printing took <8 hours and the component assembling was completed within 20 minutes. During the 3-month follow-up, the child's parents were satisfied with the prosthesis and the UNB test showed the significantly improved function of the prosthesis.

**Lessons:**

This novel 3D-printed upper limb prosthesis in a child with the traumatic wrist amputation might serve as a practical and affordable alternative for children in developing countries and those lacking access to health care providers. A personalized prosthetic rehabilitation needs to be undertaken and more clinical studies are warranted to validate the potential superiority of similar 3D-printed prostheses.

## Abstract 2

Supplemental Digital Content is available in the text

## 1 Introduction

Traumatic amputation of the upper extremity is a catastrophic event and frequently occurs in the children and young productive population. The specific injury may ultimately determine the level of amputation, it is crucial for the surgeon to consider the ultimate size, durability, and appearance of the residual limb, which will affect the ultimate satisfaction of the patient.^[1]^ Nearly 2 million Americans have lost at least one limb according to the Amputee Coalition.^[2]^ The cost of a prosthetic arm varies by the type of arm and the level of amputation. In the United States, a cosmetic arm or hand might cost $3000 to $5000, and a myoelectric prosthetic arm with realistic-looking, functioning hand costs even >$20,000.^[3]^ In developing countries, the prohibitive cost of a custom-made prosthesis brings an insufferable financial burden for the amputee's family, especially when fitting it to the growing children.^[4]^ In China, the latest China National Sample Survey on Disability (2006) showed that traumatic upper limb amputations were prevalent in rural populations and involved approximately 20 million Chinese people.^[5]^ Hence, the customizable, lightweight, and affordable upper limb prostheses are of great necessity, especially for the children with the traumatic limb deficiency.

Three-dimensional (3D) printing, also known as additive manufacturing, applies progressively in a variety of industries, including aerospace, automotive, and, notably, healthcare.^[6,7]^ Recently, advances in 3D printing are transforming to the field of prostheses with the potential to increase the accessibility, customization, and procurement of 3D-printed prostheses.^[8]^ Despite a rapid increase of the recipients of these 3D-printed hands or arms, to date, there has no practical detailed instruction existing regarding the prosthesis installation and periprosthetic rehabilitation in the growing children.

We herein report a clinical case with detailed instruction of employing a novel 3D-printed prosthesis in a child after the traumatic wrist amputation, to our very best knowledge, as the first comprehensive clinical guide for the application of the 3D-printed prosthesis. Besides, we present the short-term function outcomes of this 3D-printed prosthesis following an individualized periprosthetic rehabilitation.

## 2 Case report

### 2.1 Patient information and surgery

**Figure 1:**
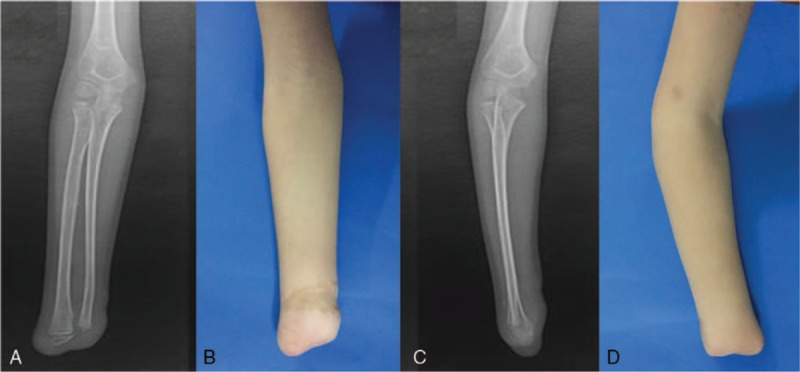
Radiographic and macroscopic examinations (anteroposterior: A, B; lateral: C, D) of the affected limb after a wrist disarticulation of the 8-year-old child.

An 8-year-old boy was referred to our department with a severe acute mangled injury of his right (dominant) hand immediately after a mincing machine accident. Severed deformity at the level of the right wrist, extensive soft tissue crush and detachment, and open wrist fracture were noted. Comminuted fracture of the bones of his right wrist was diagnosed on the emergency radiographs with a Mangled Extremity Severity Score (MESS) of 8.^[9]^ No special medical history and comorbidities needed to be addressed. A wrist disarticulation was performed as previously described.^[10]^ The key steps of the disarticulation procedure included: designing the long volar flap and the short dorsal flap; clamping, ligating, and dissecting the ulnar artery and the radial artery in the proximal radiocarpal joint; isolating and dissecting the median nerve, radial nerve, and ulnar nerve; dissecting the remaining tendons; excising the radial and ulnar styloid process; and filing the bone to smooth the contour and suturing the flap interruptly. The wound infection was found in the fifth day after the first surgery with the *Enterobacter cloacae* detected in the wound secretion. Empirical use of antibiotics and standardized wound care were applied until no bacterial growth identified by the repeated culture of the wound exudate. The patient then received a repair of the amputation stump again with the double-pedicle advancement flap. At 4 weeks after the second surgery, the wound healed successfully with stitches removed (Fig. 1). This case report was approved by the Medical Ethical Committee of Zhaoqing First People's Hospital, Guangdong, People's Republic China. The patient and the parents provided the informed consent for the publication of the clinical and radiological data.

### 2.2 Prosthesis design and manufacturing

**Figure 2:**
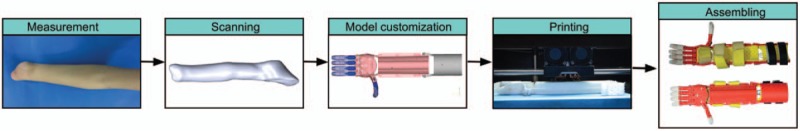
Manufacturing workflow of the 3D-printed upper limb prosthesis. The entire process includes: measurement and scanning of the limb stump, customization, printing, and assembling of the components. 3D = 3-dimensional.

**Figure 3:**
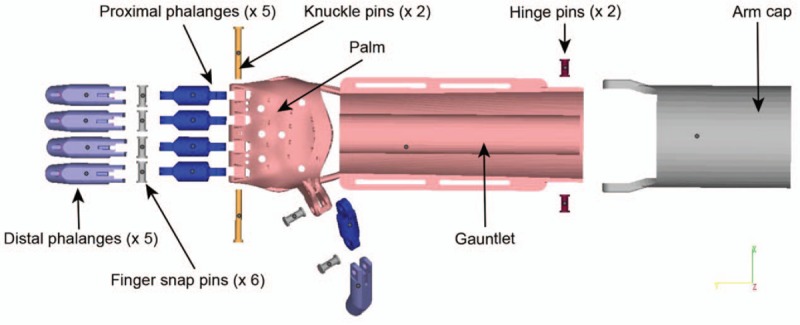
Components of the 3D-printed upper limb prosthesis. The terminal device is operated by the flexion–extension of the elbow joint with the control-cable system (not shown here). 3D = three-dimensional.

With informed consent to the patient and the caregivers about the potential benefits and harms, we designed and manufactured a novel 3D-printed prosthetic hand for this child. The design of the prosthesis was based on an open-source design (Raptor Reloaded) from the thingiverse.com and recustomized to fit the recipient.^[11]^ The whole process including: measurement and scanning of the stump, customization, printing, and assembling of the components (Fig. 2). The length and circumference of both the unaffected arm and the residual part of the affected arm of the recipient were measured as a reference for the customization of the prosthesis. The data was then managed with Slic3r (slic3r.org) and each component of the prosthesis was reshaped individually. Components of the device included distal phalanges, proximal phalanges, finger snap pins, knuckle pins, palm, gauntlet (forearm), hinge pins, and arm cap (Fig. 3). Each component was fabricated with an open sourced 3D printer (FlashForge Creator Pro, Zhejiang, China) using the acrylonitrile–butadiene–styrene filament. The components were then assembled with the nylon string and the medical-level foam. The control–cable system allowed the distal phalanges to open with the extension and to close with the flexion of the wrist of the recipient.

### 2.3 Periprosthetic rehabilitation

The personalized prosthetic training and rehabilitation program were applied in this recipient after the installation of the device at 6 months after the second surgery.^[12]^ The program included preprosthetic, interim, and postprosthetic training (Table 1). In the preprosthetic period, care was given to keep the normal range of motion and strengthening of the amputated arm, shape the stump for fitting the prosthesis, and take care of the scar and edema. After fitting of the prosthesis, the amputee received the scheduled training for prosthetic control, repetitive drills, and activities of daily living (ADL). The ADL training was performed according to the rating guide developed by Atkin's titled “Unilateral Upper Extremity Amputation—Activities of Daily Living”.^[13]^ The patient was asked to visit the outpatient physical therapist twice per week for the first 8 weeks, once per week for the next 4 weeks, and then once per month within the first year. Education of both the recipient and the parents was also provided, involving the loading, cleaning, and supervision of the prosthesis in the daily activities.

**Table 1:**
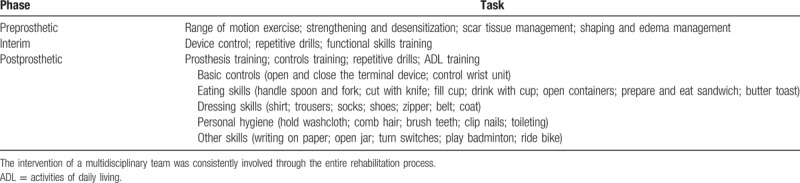
Phases of the prosthetic training and periprosthetic rehabilitation.

### 2.4 Follow-up and prosthesis evaluation

The function evaluation of the prosthesis was performed at 1 month and 3 months postprosthetically. The Children Amputee Prosthetics Projects (CAPP) score^[14,15]^ and The University of New Brunswick Test of Prosthetic Function for Unilateral Amputees (UNB test)^[16]^ were used to assess the daily functionality of the prosthesis, which were the standardized tools with previously validated reliability. The CAPP score includes the CAPP-Prosthesis Satisfaction Inventory (CAPP-PSI)^[14]^ and the CAPP-Functional Status Inventory (CAPP-FSI),^[15]^ and both are required to be completed by the child's parents. The CAPP-PSI measures the degree of parents’ satisfaction with their child's prosthetic hand regarding fit, function, appearance, and service (see Supplementary Table S1, which illustrates the entire 14 items of the CAPP-PSI). Each item is evaluated using a 4-point score ranging from 0 to 4 (0 = not at all”; 1 = a little; 2 = somewhat; 3 = a lot; and 4 = very much). The CAPP-FSI measures the child's performance of daily behaviors including self-care tasks and other developmentally activities. The child's behavior is rated on 2 scales: “does activity” indicates the frequency of the child independently performing a specific task, and “Uses prosthesis” signifies the frequency of the child using prosthesis to perform the tasks (see Supplementary Table S2, which illustrates the entire 34 items of the CAPP-FSI for children with upper limb deficiency). Each item was rated on a 5-point score ranging from 0 to 5 (0 = none of the time, 1 = a little of time, 2 = some of the time, 3 = most of the time, and 4 = all the time). The UNB test for children (ages 8–10) includes 3 subtests (10 items per subtest) which demonstrated intersubtest reliability (see Supplementary Tables S3–S6, which illustrate the 3 subtests and the rating scale of the UNB test). It evaluates 2 scales (spontaneity and skill) of the utilization of the prosthesis. Performance of each testing maneuver was video recorded and evaluated subsequently with a 5-point scale (0–4) for both the spontaneity and skill of the utilization of the prosthesis.

The results were analyzed by 2 independent observers (GSX and LG) and reported as a consensus with discussion. Descriptive statistics were used to report the scores. Data was shown in mean ± standard deviation. The mean score of the UNB test (from 30 items of 3 subtests) at 1 month and 3 months postprosthetically were compared using the Mann–Whitney U test with a significance level of .05.

## 3 Outcomes

**Figure 4:**
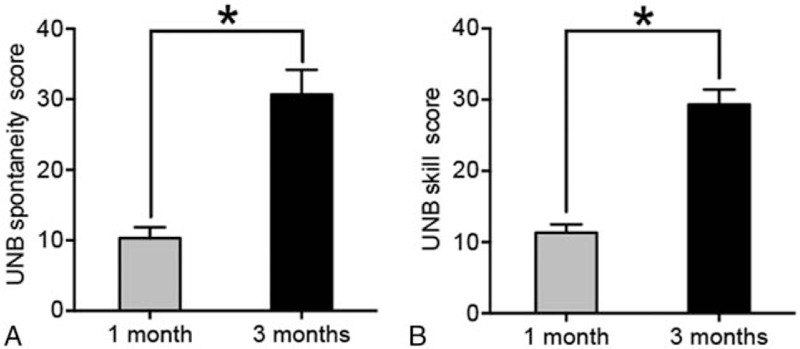
The UNB spontaneity (A) and skill (B) scores showing the significantly improved function of the prosthesis following the scheduled training at 3 months postprosthetically. ^∗^*P *<.001. UNB test = University Of New Brunswick Test Of Prosthetic Function for Unilateral Amputees.

All the materials cost <$20. The entire printing process took <8 hours, and the final component assembling was completed within 20 minutes. After 3-month follow-up with standardized rehabilitation, the child's parents were satisfied with the prosthesis with a total CAPP-PSI score of 35 and a CAPP-FSI score of 28. The UNB spontaneity score improved from 10.33 ± 1.53 at 1 month to 30.67 ± 3.51 at 3 months postprosthetically (*P* <.001), and the UNB skill score increased from 11.33 ± 1.16 at 1 month to 29.33 ± 2.08 at 3 months postprosthetically (*P* <.001) (Fig. 4). The child functioned satisfactory in various activities of daily living, such as eating, writing, self-dressing, and riding bicycle, when utilizing the prosthesis (see Supplemental Video, which representatively demonstrates the functionality of the prosthesis in activities of daily living). Some fingers, knuckles, and cables were replaced twice due to the daily wear and tear. Both the recipient and the parents did not report any special complications regarding to this 3D-printed prosthesis.

## 4 Discussion

With advances of 3D printing and increasing number of household 3D printers, 3D-printed upper extremity prostheses are emerging as affordable, lightweight, and customizable alternatives to traditional orthotist-produced prostheses. We described a clinical case with detailed instruction of employing a novel 3D-printed prosthesis in a child after the trauma-related wrist disarticulation. The short-term function evaluation showed that this novel 3D-printed device was satisfactory and practical with an improved function following a personalized periprosthetic rehabilitation.

In the present study, we demonstrated this novel 3D-printed prosthetic hand is cost-effective, customized, well-fitting with a considerable practical value. Both the UNB spontaneity score and the UNB skill score of the device improved significantly following the scheduled training and rehabilitation, indicating the sound functionality of this 3D-printed prosthesis. Notable advantages of the 3D-printed prosthesis include lower cost compared with the conventional prosthesis and easy customization for recipients to print and replace parts by themselves. The easy accessibility of open-source design files shared online and the recent influx of house-use 3D printer allows people to create specific device for each activity, interest, and size. Zuniga et al^[17]^ described a Cyborg Beast 3D-printed device and proposed it as a possible low-cost alternative for children in developing countries and those with little or no access to health-care providers. The limitations of these 3D-printed prostheses also need to be underlined. First, most currently available open-source designs are not regulated or verified by the health-care professionals, and the potential unrevealed harms should not be neglected. Second, although the prosthesis was applicable to specific tasks, for example, riding bike or playing badminton, it still functioned awkwardly in certain fine or bimanual movements, such as tying shoelaces or making pipe cleaner sculpture, even after the scheduled training and rehabilitation for 3 months postprosthetically. These data might underline that a more sophisticated design and advanced prosthesis training are needed to ensure the better clinical outcomes.

Of note, the multidisciplinary teamwork of physicians, surgeons, physical therapists, orthotics, and physiologists, and parents (caregivers) is critical to ensure a successful performance of this 3D-printed prosthesis in the children amputee.^[18]^ The early and deep involvement of the physical therapist is highly recommended. The training program should be formulated individually according to the level of limb loss and the design of the prosthesis. Caregiver vigilance is also vital to ensure the devices fit properly and to prevent device-related distress or fatigue.

There are currently dozens of hand and arm model designs available to address a multitude of upper limb deficiency, ranging from single finger loss to full arm absences. Despite a daily increase in the production of these noncommercially devices by nonmedical professionals, clinical guidelines of patient and device selection and official approval on the manufacture and distribution of these devices are still lacking.^[8]^ Elder children aged over 5 year might enable more effective adherence to the training order and reliable reporting the functionality of the device. More studies are still warranted to assess the potential disadvantages of these 3D-printed prostheses and to compare them with conventional custom-made prostheses. The reception, function, and quality of life among different designs also need to be explored by studies with long-term follow-up.

Overall, this clinical case with detailed instruction of applying a novel 3D-printed upper limb prosthesis in a child with the trauma-related wrist disarticulation presents that this 3D-printed prosthesis might serve as a practical and affordable alternative for children in developing countries and those lacking access to health care providers. A personalized prosthetic rehabilitation needs to be undertaken and more clinical studies are warranted to validate the potential superiority of similar 3D-printed prostheses.

## Supplementary Material

Supplemental Digital Content

## Supplementary Material

Supplemental Digital Content
