# Supplementary material for: Three-dimensional-printed upper limb prosthesis for a child with traumatic amputation of right wrist: A case report
Source: Medicine (Baltimore). 2017 Dec 29;96(52):e9426. doi: 10.1097/MD.0000000000009426 (PMC6392638; doi:10.1097/MD.0000000000009426)
Supplement: Supplemental Digital Content [file medi-96-e9426-s002.doc]

**Table S1.** Child amputee prosthetics projects – prosthesis satisfaction inventory (CAPP-PSI).

| Item | Description |
| --- | --- |
|  | (Parent-rated) child satisfaction with prosthesis |
| 1 | Aids in daily activities |
| 2 | Fits |
| 3 | Functions |
| 4 | Appearance |
|  | Parent satisfaction with prosthesis |
| 5 | Aids in daily activities |
| 6 | Fits |
| 7 | Functions |
| 8 | Appearance |
|  | Parent satisfaction with service |
| 9 | With evaluation on delivery |
| 10 | With follow-up care |
| 11 | With instruction |
| 12 | With manufacture time |
| 13 | With repair time |
| 14 | With child’s training |

The child’s parents select a response for each item using a 5-point score ranging from 0 to 4 (0 = ‘‘not at all’’; 1 = ‘‘a little’’; 2 = ‘‘somewhat’’; 3 = ‘‘a lot’’; 4 = ‘‘very much’’).

**Table S2**. Child amputee prosthetics projects – prosthesis functional status inventory (CAPP-FSI).

| Item | Description |
| --- | --- |
| 1 | Fold a large towel |
|  | Does Activity |
|  | Uses Prosthesis |
| 2 | Put toothpaste on brush and brush teeth |
|  | Does Activity |
|  | Uses Prosthesis |
| 3 | Hold a blow dryer and style hair with a brush |
|  | Does Activity |
|  | Uses Prosthesis |
| 4 | Put on and remove a T-shirt over the head |
|  | Does Activity |
|  | Uses Prosthesis |
| 5 | Put on and remove a shirt with front buttons |
|  | Does Activity |
|  | Uses Prosthesis |
| 6 | Button and unbutton shirt or blouse |
|  | Does Activity |
|  | Uses Prosthesis |
| 7 | Buckle and unbuckle a belt |
|  | Does Activity |
|  | Uses Prosthesis |
| 8 | Attach the ends of a zipper and zip a jacket |
|  | Does Activity |
|  | Uses Prosthesis |
| 9 | Put on and remove socks |
|  | Does Activity |
|  | Uses Prosthesis |
| 10 | Put on and remove shoes |
|  | Does Activity |
|  | Uses Prosthesis |
| 11 | Tie shoe laces |
|  | Does Activity |
|  | Uses Prosthesis |
| 12 | Cut food with a knife and fork |
|  | Does Activity |
|  | Uses Prosthesis |
| 13 | Peel a potato or apple with a peeler or knife |
|  | Does Activity |
|  | Uses Prosthesis |
| 14 | Lift glass/cup to mouth and drink |
|  | Does Activity |
|  | Uses Prosthesis |
| 15 | Open a jar which has already been opened |
|  | Does Activity |
|  | Uses Prosthesis |
| 16 | Open a pack of candy or gum |
|  | Does Activity |
|  | Uses Prosthesis |
| 17 | Open a sealed bag, like potato chips, that has not already been opened |
|  | Does Activity |
|  | Uses Prosthesis |
| 18 | Hold a soft drink can and open a tab-top |
|  | Does Activity |
|  | Uses Prosthesis |
| 19 | Hold a sandwich and eat |
|  | Does Activity |
|  | Uses Prosthesis |
| 20 | Close a plastic sandwich bag with a twist-tie |
|  | Does Activity |
|  | Uses Prosthesis |
| 21 | Hold a bowel ad mix a sauce or batter |
|  | Does Activity |
|  | Uses Prosthesis |
| 22 | Slice a piece of fruit, vegetable, or bread |
|  | Does Activity |
|  | Uses Prosthesis |
| 23 | Roll out cookie dough with a rolling pin |
|  | Does Activity |
|  | Uses Prosthesis |
| 24 | Turn pages in a book |
|  | Does Activity |
|  | Uses Prosthesis |
| 25 | Hold paper and cut with a scissor |
|  | Does Activity |
|  | Uses Prosthesis |
| 26 | Open a flashlight and change batteries |
|  | Does Activity |
|  | Uses Prosthesis |
| 27 | Hold a hand of cards and play a game |
|  | Does Activity |
|  | Uses Prosthesis |
| 28 | Assemble a construction-type toy (eg, legos, duplo, nuts and bolts, etc) |
|  | Does Activity |
|  | Uses Prosthesis |
| 29 | Wind up a wind-up toy or an alarm clock |
|  | Does Activity |
|  | Uses Prosthesis |
| 30 | Thread a needle |
|  | Does Activity |
|  | Uses Prosthesis |
| 31 | Hammer a nail into a piece of wood |
|  | Does Activity |
|  | Uses Prosthesis |
| 32 | Build a model airplane or car |
|  | Does Activity |
|  | Uses Prosthesis |
| 33 | Sew on a button |
|  | Does Activity |
|  | Uses Prosthesis |
| 34 | Put a key on a key chain |
|  | Does Activity |
|  | Uses Prosthesis |

The CAPP-FSI is rated on two scales: ‘’Does activity’’ and ‘’Uses prosthesis’’. For the upper limb deficiency, each one of the total 34 items is rated on a 5-point scale ranging from 0 to 4 (0= ‘’none of the time’’, 1 = ‘’a little of time’’, 2 = ‘’some of the time’’, 3 = ‘’most of the time’’, and 4 = ‘’all the time’’).

**Table S3**. Subtest #1 of the University of New Brunswick (UNB) test of Prosthetics Function for children (ages 8-10) with unilateral upper limb deficiency.

| Item | Description |
| --- | --- |
| 1 | Item: start a zipper (the kind that separates at the bottom)  Equipment: provide jacket big enough for child to put on, with large, easy zipper and large pull tab  Score: use of prosthesis to stabilize one side of zipper  Special Instructions: N/A |
| 2 | Item: hang jacket on coat hanger  Equipment: jacket from #1; wire coat hanger  Score: method of holding jacket and hanger  Special Instructions: N/A |
| 3 | Item: open box of blackboard chalk and remove a piece  Equipment: a closed flip-top box of chalk  Score: method of stabilizing box  Special Instructions: N/A |
| 4 | Item: draw on blackboard while holding notebook  Equipment: notebook with pictures, phrases, jokes, etc. to copy; chalk from #3  Score: method of holding notebook and chalk  Special Instructions: N/A |
| 5 | Item: play with a yo-yo  Equipment: small yo-yo that can be grasped around circumference by the terminal device  Score: method of holding yo-yo to rewind string  Special Instructions: N/A |
| 6 | Item: use paper punch  Equipment: single hole paper punch; sheet of paper  Score: method of stabilizing paper to punch holes  Special Instructions: N/A |
| 7 | Item: seal polythene bag with twist tie  Equipment: one small polythene bag with strip ' of wire twist ties; item to put in bag  Score: method of securing wire tie around bag  Special Instructions: N/A |
| 8 | Item: play card game  Equipment: hand of five to seven playing cards  Score: method of holding hand of cards to play  Special Instructions: N/A |
| 9 | Item: open a plastic "bubble"  Equipment: foil-sealed bubble of jam or butter  Score: method of stabilizing plastic container to peel off foil  Special Instructions: N/A |
| 10 | Item: spread jam on crackers  Equipment: jam or butter from #9, crackers, knife  Score: method of stabilizing crackers and knife  Special Instructions: N/A |

The UNB test for children (ages 8-10) were used including 3 subtests (10 items per subtest) with demonstrated inter-subtest reliability. It evaluates two scales (spontaneity and skill) of the utilization of the prosthesis. Performance of each testing maneuver was video recorded and evaluated subsequently with a 5-point scale (0-4) for both the spontaneity and skill of the utilization of the prosthesis. Please refer to Table S6 for more details of the rating scale of the UNB test.

**Table S4.** Subtest #2 of the University of New Brunswick (UNB) test of Prosthetics Function for children (ages 8-10) with unilateral upper limb deficiency.

| Item | Description of maneuver |
| --- | --- |
| 1 | Item: hit ball with bat  Equipment: bat normally used for baseball, rounders or cricket; ball  Score: method of holding bat  Special Instructions: ball could be larger than one normally used |
| 2 | Item: pin badge on shirt  Equipment: metal badge with straight pin fastener on back; T-shirt placed on table in front of child  Score: method of stabilizing shirt to attach badge  Special Instructions: instruct child to pin badge to one layer of fabric only. |
| 3 | Item: fold shirt  Equipment: T-shirt from #2, on table in front of child  Score: method of folding shirt  Special Instructions: N/A |
| 4 | Item: open bandaid and apply  Equipment: paper-wrapped bandaid strip  Score: method of opening bandaid and tearing off adhesive protectors  Special Instructions: N/A |
| 5 | Item: take off jacket or cardigan  Equipment: provide jacket or cardigan if necessary  Score: attempt to grasp cuff of clothing on sound side with terminal device  Special Instructions: sleeves must be close-fitting, so jacket cannot be shrugged off |
| 6 | Item: ball and blanket game  Equipment: small blanket (baby's receiving blanket) or large towel; large light ball  Score: method of holding blanket  Special Instructions: two players stand facing each other. Each holds two corners of blanket. Ball is placed on blanket and bounced. Object is to try to keep ball aloft |
| 7 | Item: make a "telescope"  Equipment: one sheet of typing paper; one rubber band  Score: method of holding rolled paper to slip on the rubber band  Special Instructions: N/A |
| 8 | Item: fill paper cup with water  Equipment: cone shaped cup; water faucet  Score: method of filling cup  Special Instructions: cone shaped cup prevents child from setting it on the bottom of the sink. Use of prosthesis to either hold cup or turn faucet is scored. |
| 9 | Item: open jar  Equipment: glass jar with twist lid no larger than 1 3/4" (4cm) diameter, with small toy (or candy from #10) inside  Score: method of removing lid  Special Instructions: N/A |
| 10 | Item: unwrap a candy with close fitting wrapper  Equipment: cellophane wrapped caramel or blackjack  Score: method of removing wrapper  Special Instructions: N/A |

The UNB test for children (ages 8-10) were used including 3 subtests (10 items per subtest) with demonstrated inter-subtest reliability. It evaluates two scales (spontaneity and skill) of the utilization of the prosthesis. Performance of each testing maneuver was video recorded and evaluated subsequently with a 5-point scale (0-4) for both the spontaneity and skill of the utilization of the prosthesis. Please refer to Table S6 for more details of the rating scale of the UNB test.

**Table S5.** Subtest #3 of the University of New Brunswick (UNB) test of Prosthetics Function for children (ages 8-10) with unilateral upper limb deficiency.

| Item | Description of maneuver |
| --- | --- |
| 1 | Item: tie a scarf around neck  Equipment: rectangular scarf (wool or silk), approximately 3 feet (1 meter) long  Score: method of tying scarf  Special Instructions: N/A |
| 2 | Item: pull glove onto sound hand  Equipment: pair of gloves  Score: method of donning glove  Special Instructions: can be any glove appropriate to another activity, (eg. white glove for dressing up as clown, gardening glove, child's own glove, etc.) |
| 3 | Item: tie shoelaces  Equipment: sneaker with laces of adequate length  Score: method of tying laces, knot and bow  Special Instructions: child's own sneaker preferable, or supply one if necessary - score use of prosthesis, not ability to tie laces |
| 4 | Item: serve in badminton game  Equipment: badminton racquets and bird  Score: method of serving  Special Instructions: N/A |
| 5 | Item: make pipe cleaner sculpture  Equipment: package of pipe cleaners (coloured if available)  Score: method of stabilizing pipe cleaners to twist together  Special Instructions: N/A |
| 6 | Item: lift end of table and move it  Equipment: small table  Score: use of prosthesis to assist in lifting table  Special Instructions: examiner lifts other end of table |
| 7 | Item: spread table cloth on table  Equipment: folded table cloth; table  Score: method of handling cloth  Special Instructions: N/A |
| 8 | Item: remove facial tissue from purse-size package  Equipment: one plastic or paper purse-size package of tissues with perforation down the centre  Score: method of removing tissue from package  Special Instructions: package is not necessarily unopened |
| 9 | Item: tear masking tape off roll  Equipment: 1 roll of 1" masking tape  Score: method of stabilizing roll while pulling and tearing tape  Special Instructions: N/A |
| 10 | Item: open a package of potato chips  Equipment: small bag of potato chips (foil or cellophane)  Score: method of opening package  Special Instructions: N/A |

The UNB test for children (ages 8-10) were used including 3 subtests (10 items per subtest) with demonstrated inter-subtest reliability. It evaluates two scales (spontaneity and skill) of the utilization of the prosthesis. Performance of each testing maneuver was video recorded and evaluated subsequently with a 5-point score (0-4) for both the spontaneity and skill of the utilization of the prosthesis. Please refer to Table S6 for more details of the rating scale of the UNB test.

**Table S6.** Rating scale of the University of New Brunswick (UNB) test of Prosthetics Function for children (ages 8-10) with unilateral upper limb deficiency.

| Index | Score |
| --- | --- |
| Spontaneity of Prosthetics Function |  |
| Immediate, automatic, consistent use of terminal device for active grasp | 4 |
| Slightly delayed or inconsistent use of terminal device for active grasp | 3 |
| Very delayed, occasional or "last resort" use of the terminal device (either for active grasp or passively) | 2 |
| Use of prosthesis proximal to terminal device only | 1 |
| Prosthesis not used or used only on request | 0 |
| Skill of Prosthetics Function |  |
| Active use of terminal device is quick, skilled and smooth. Grasp is consistently maintained | 4 |
| Active use of terminal device, shows some degree of awkwardness, slowness or uncertainty, Grasp is readily regained when lost | 3 |
| Active use of terminal device is attempted, but looks very slow or awkward. Grasp is frequently lost or regained with difficulty. | 2 |
| No active terminal device function, although terminal device or some other part of prosthesis may be used passively to stabilize or support. | 1 |
| Prosthesis not used | 0 |

The rating scale for the UNB Test of Prosthetics Function requires the examiner to observe two distinct aspects of prosthesis use: spontaneity and skill. Both are measured on a 5 point scale from 0 to 4. The term "active" refers to the grasping and holding functions of the terminal device. "Passive" describes use of the prosthesis as a "paper weight", without any attempt to open or close the terminal device. The Spontaneity index measures the extent to which the child has incorporated the prosthesis into his or her body image. The examiner must observe the child's tendency to use the prosthesis to assist with the task. Judgment of the automatic use of the prosthesis is a unique feature of the UNB Test and examiners must be careful to distinguish between the Spontaneity and Skill indices of the Rating Scale. The Skill index measures the aspects of prosthesis function that most therapists are accustomed to observing, that is, the ability to open and close the terminal device to grasp and release objects of different sizes and shapes with confidence, speed and consistency. As well, it includes the ability to maintain grasp without letting go accidentally, the ability to apply the correct amount of pressure and the awareness of how wide to open the terminal device. All of these skills contribute to the dexterity with which the prosthesis is used.
